# The impact of a multifaceted intervention including sepsis electronic alert system and sepsis response team on the outcomes of patients with sepsis and septic shock

**DOI:** 10.1186/s13613-017-0280-7

**Published:** 2017-05-30

**Authors:** Yaseen M. Arabi, Hasan M. Al-Dorzi, Ahmed Alamry, Ra’ed Hijazi, Sami Alsolamy, Majid Al Salamah, Hani M. Tamim, Saad Al-Qahtani, Abdulaziz Al-Dawood, Abdellatif M. Marini, Fatimah H. Al Ehnidi, Shihab Mundekkadan, Amal Matroud, Mohamed S. Mohamed, Saadi Taher

**Affiliations:** 1 Intensive Care Department, MC-1425, Respiratory Services, College of Medicine, King Abdullah International Medical Research Center, King Saud bin Abdulaziz University for Health Sciences, P.O. Box 22490, Riyadh, 11426 Kingdom of Saudi Arabia; 2 King Abdullah International Medical Research Center, King Saud bin Abdulaziz University for Health Sciences, Riyadh, Kingdom of Saudi Arabia; 3 Department of Internal Medicine, American University of Beirut- Medical Center, Beirut, Lebanon

**Keywords:** Sepsis, Shock, Intensive care unit, Hospital mortality, Quality improvement, Patient safety, Health service administration, Emergency department, Sepsis resuscitation bundle

## Abstract

**Background:**

Compliance with the clinical practice guidelines of sepsis management has been low. The objective of our study was to describe the results of implementing a multifaceted intervention including an electronic alert (e-alert) with a sepsis response team (SRT) on the outcome of patients with sepsis and septic shock presenting to the emergency department.

**Methods:**

This was a pre–post two-phased implementation study that consisted of a pre-intervention phase (January 01, 2011–September 24, 2012), intervention phase I (multifaceted intervention including e-alert, from September 25, 2012–March 03, 2013) and intervention phase II when SRT was added (March 04, 2013–October 30, 2013) in a 900-bed tertiary-care academic hospital. We recorded baseline characteristics and processes of care in adult patients presenting with sepsis or septic shock. The primary outcome measures were hospital mortality. Secondary outcomes were the need for mechanical ventilation and length of stay in the intensive unit and in the hospital.

**Results:**

After implementing the multifaceted intervention including e-alert and SRT, cases were identified with less severe clinical and laboratory abnormalities and the processes of care improved. When adjusted to propensity score, the interventions were associated with reduction in hospital mortality [for intervention phase II compared to pre-intervention: adjusted odds ratio (aOR) 0.71, 95% CI 0.58–0.85, *p* = 0.003], reduction in the need for mechanical ventilation (aOR 0.45, 95% CI 0.37–0.55, *p* < 0.0001) and reduction in ICU LOS and hospital LOS for all patients as well as ICU LOS for survivors.

**Conclusions:**

Implementing a multifaceted intervention including sepsis e-alert with SRT was associated with earlier identification of sepsis, increase in compliance with sepsis resuscitation bundle and reduction in the need for mechanical ventilation and reduction in hospital mortality and LOS.

**Electronic supplementary material:**

The online version of this article (doi:10.1186/s13613-017-0280-7) contains supplementary material, which is available to authorized users.

## Background

Despite the major burden of sepsis as a leading cause of human death, studies have repeatedly demonstrated that the compliance with the clinical practice guidelines for sepsis management, grouped in the sepsis resuscitation bundle, is low and that this low compliance is associated with increased mortality [1–9]. As a result, the Surviving Sepsis Campaign (SSC) has recommended performance improvement efforts be undertaken [10, 11] and the National Quality Forum has endorsed the bundle implementation [12]. Thus, several institutional, national and international improvement projects have been launched [13–16].

However, transitioning evidence into sustainable clinical improvement in sepsis management has been a complex task [17–19]. Implementation barriers include delayed identification of septic patients, unawareness of or disagreement with guidelines, guideline complexity, lack of resources [17–19], staff shortage and unavailability of specialized setting required to put therapy into routine practice [9]. In the face of this complexity, different implementation strategies have been utilized, including education, posters, reminders, audit and feedback, paper-based and electronic sepsis screening tools, clinical pathways, rapid response teams and sepsis response teams (SRTs) [20–28]. The differential or the synergistic effect of these strategies on changing sepsis management practice has not been studied. However, it has been shown in other settings that these interventions vary in effectiveness. Two systematic reviews showed that educational activities and audits had low leverage on changing physician practices and reminders had a slightly larger effect [29, 30]. Automation and forcing function have higher leverage [31], and a multifaceted approach may be the most effective [29, 30]. Yet, many sepsis improvement projects invested in potentially low-leverage interventions leading to modest change in sepsis bundle compliance and mortality. The use of automation and forced function in sepsis management may be achieved by combining an electronic sepsis alert (e-alert) system for timely sepsis identification with a sepsis response team for timely management. Although there are a few reports on implementing these two interventions individually [32–34], the inclusion of the two as components of a multifaceted intervention, which may have a synergistic effect [26], has not been studied.

The objective of this study was to examine the impact of an improvement project utilizing a multifaceted intervention that includes an e-alert system and SRT on the compliance with the sepsis resuscitation bundle and outcome of adult patients with sepsis and septic shock presenting to the emergency department (ED).

## Methods

### Setting

The study was conducted in a 900-bed tertiary-care academic hospital accredited by Joint Commission International. The ED receives >200,000 patients per year is staffed by board-certified emergency medicine physicians and has 15 resuscitation beds for high-acuity patients and 49 beds for moderate-acuity patients. The Intensive Care Department staffs 5 ICUs on a 24-h/7-day in-house basis [35], has a rapid response team [36] and provides coverage for boarding patients in the ED who meet ICU admission criteria.

### Study design

This was a pre–post implementation study that consisted of a pre-intervention phase and a 2-step implementation (intervention phases I and II). During the study period, there were no major changes in the hospital admission criteria, nursing or medical care plans, or staffing structure. The study was approved by the Institutional Review Board of Ministry of National Guard-Health affairs, and informed consent requirement was waived.

### Pre-intervention phase

In this phase (January 01, 2011–September 24, 2012), patients presenting to the ED were triaged based on illness severity according to the Canadian Triage and Acuity Scale [37], evaluated first by the ED nurses and physicians, and then referred to a primary admitting service. Critically ill septic patients were subsequently referred to the intensive care team. Identifying patient as having sepsis and initial resuscitation were based on clinical assessment by the ED physicians, the primary admitting service, the intensive care team or a combination of these services. During this phase, the sepsis bundle had not been implemented.

### Intervention phase I

In this phase (September 25, 2012–March 03, 2013), sepsis e-alert and computerized physician order entry (CPOE) sepsis management order-set (based on the SCC sepsis bundle) were implemented and were accompanied by an educational campaign targeting the ED healthcare providers. A clinical pathway was also generated after multidisciplinary discussions (Additional file 1: Figure S1, Figure S2, Table S1). Additionally, weekly text messages were sent to the phones of the ED and ICU physicians about the bundle compliance rates highlighting the element of the bundle that had the lowest compliance.

This phase required considerable planning, which started in October 2011 as a multidisciplinary quality improvement project aimed at improving sepsis management through the implementation of the SSC bundle. Root-cause analysis was conducted to search for the probable causes of low bundle compliance. In addition, the flow of septic patients from the ED to the ICU was mapped to understand related processes and bottlenecks. After analysis, the low compliance was attributed to multiple factors including delays in sepsis recognition, insufficient awareness of sepsis resuscitation bundle and the complexity of the care processes. For the intervention phase I, the project focused on improving early sepsis recognition through building an electronic sepsis screening tool (e-alert) in the hospital electronic health record (EHR) system (QuadraMed^®^ Computerized Patient Record System, Reston, VA, USA) and on simplifying the care process by building a CPOE order-set that incorporated the required orders in groups on one platform. The e-alert was based on identifying abnormal vital signs and certain laboratory tests, all captured from the EHR. We used in designing our e-alert the formal definition of sepsis (previously called severe sepsis) with includes a combination of the Systemic Inflammatory Response Syndrome (SIRS) criteria and one organ dysfunction, because this quality improvement project was performed the new definition for sepsis (Sepsis-3) became available [38.] When a patient demonstrated any two SIRS criteria (temperature >38 °C OR <36 °C; heart rate >90 beats/min; respiratory rate >20 breaths/min; WBC count >12,000/mm^3^ OR <4000/mm^3^) AND at least one of the following organ dysfunctions (systolic blood pressure 86–90 mmHg with intravenous fluids or <86 mmHg regardless of fluids; blood oxygen saturation of 85–90% with supplemental oxygen or <85% without oxygen; lactate >2 mmol/L) OR two of the above organ dysfunctions, an e-alert was sent to the nursing work-list prompting her/him to contact the treating medical team. The treating physician would then evaluate the patient, confirm the presence or absence of these conditions and use the CPOE order-set if indicated. The development and testing of both the e-alert and the CPOE order-set went through multiple PDSA (plan, do, study, act) cycles with small-scale testing, documentation and learning in the EHR system development (in-production) domain until the activation date. During these PDSA cycles, certain triggers were added to the e-alert (systolic blood pressure 86–90 mmHg with intravenous fluids or <86 mmHg regardless of fluids; blood oxygen saturation of 85–90% with supplemental oxygen or <85% without oxygen) to improve specificity. We could not use reduced level of consciousness in building the e-alert, because it was recorded as a text and not as a numeric value in our EHR.

### Intervention phase II

On March 04, 2013, and through October 30, 2013, a dedicated 24/7 SRT was launched in addition to the interventions already in place from intervention phase I. The SRT consisted of an intensive care registrar physician and a nurse who were trained on sepsis management using didactic lectures and simulation-based learning. The bedside nurse activated the SRT after being prompted by the e-alert or when sepsis was clinically suspected. The SRT assessed the patient for the presence of sepsis, followed the same clinical pathway used in intervention phase I and provided sepsis management as needed in collaboration with the treating team.

### Data collection and outcome measures

Starting October 2011, we collected patient-level data prospectively by a dedicated data collector using the 2008 SSC data collection tool [39]. Additional data were collected retrospectively for the period January 2011–September 2011, to expand the baseline data of pre-intervention phase. Sepsis (originally severe sepsis) was defined as SIRS with acute organ dysfunction secondary to documented or suspected infection. The organ dysfunctions that were used to build the e-alert were hypotension, hypoxia and increased lactate as defined above. Septic shock was defined as sepsis (originally severe sepsis) with persistent hypotension after fluid resuscitation with at least 20 mL/kg of crystalloid (or equivalent). We recorded the compliance with the following individual elements of the 2008 SSC resuscitation bundle and the time to intervention: [1] serum lactate obtained within 6 h of presentation, [2] blood cultures taken before the administration of broad-spectrum antibiotics, [3] broad-spectrum antibiotics administered within 3 h of ED admission, [4] 20 mL/kg of crystalloid (or equivalent) administered in patients who were hypotensive or had lactate >4 mmol/L; and in patients who remained hypotensive or had lactate >4 mmol/L, [6] achievement of central venous pressure (CVP) ≥8 mmHg and central venous oxygen saturation (ScvO_2_) ≥70% and initiation of vasopressors. Based on recent evidence arising from the recent clinical trials on early goal-directed therapy [40–42], we evaluated bundle compliance with the first 4 elements only and we did not include CVP and ScvO_2_. The primary outcome measure was hospital mortality. Secondary outcome measures were the need for mechanical ventilation during this episode of sepsis, ICU and hospital length of stay (LOS) for all patients for survivors.

### Statistical analysis

All statistical analyses were performed using SAS software (version 9.1; SAS Institute, Cary, NC). Continuous variables were presented as means with standard deviations, and categorical variables as absolute and relative frequencies. Mann–Whitney and Chi-square tests were used to compare differences between the phases as appropriate. To account for the differences in baseline characteristics among the three phases, we generated propensity scores using the following variables that were clinically relevant and statistically associated with the different phases (Table 1): pneumonia, hypothermia, acutely altered mental status, hypoxia, leukopenia, increased creatinine, thrombocytopenia, hyperbilirubinemia, coagulopathy and lactate levels. To check the balancing effect of propensity scores, we used the propensity scores as covariates in an analysis of covariance for continuous baseline variables and in multinomial logistic regression for categorical baseline variables. We used multivariate logistic and linear regression analysis to examine the association of implementation phase with mortality and LOS after adjustment to propensity scores. A *p* value <0.05 was considered to be statistically significant. We constructed control charts with upper and lower control limits for the 4-element bundle compliance and for hospital mortality using CHARTrunner, version 3.6.88 (PQ Systems, Dayton OH, USA).

**Table 1:** Baseline characteristics of patients with sepsis and septic shock on presentation in the three study phases: pre-intervention (A), intervention phase I (B) and intervention phase II (C)

All patients	Pre-intervention A	Intervention phase I B	Intervention phase II C	Crude *p* value			*p* value after propensity score adjustment		
	*N* = 436	*N* = 195	*N* = 699	B vs. A	C vs. B	C vs. A	B vs. A	C vs. B	C vs. A
Source of sepsis, no. (%)									
Pneumonia	228 (52.3)	93 (47.7)	281 (40.2)	0.29	0.06	<0.0001	0.99	0.98	0.96
Urinary tract infection	70 (16.1)	28 (14.4)	107 (15.3)	0.59	0.74	0.74	0.78	0.90	0.38
Acute abdominal infection	40 (9.2)	8 (3.6)	47 (6.7)	0.03	0.18	0.13	0.05	0.37	0.17
Soft tissue infection	8 (1.8)	8 (4.1)	32 (4.6)	0.10	0.78	0.01	0.42	0.84	0.56
Other infections	138 (31.7)	68 (34.9)	254 (36.3)	0.43	0.71	0.11	0.92	0.60	0.48
Signs and symptoms, no. (%)									
Hyperthermia^a^	114 (26.2)	52 (26.7)	172 (24.6)	0.89	0.56	0.56	0.81	0.15	0.47
Hypothermia^a^	34 (7.8)	6 (3.1)	7 (1.0)	0.02	0.04	<0.0001	0.99	0.86	0.81
Acutely altered mental status	157 (36.0)	48 (24.6)	59 (8.4)	0.005	<0.0001	<0.0001	0.98	0.67	0.71
Chills and rigors	11 (2.5)	7 (3.6)	2 (0.3)	0.46	0.001	0.0006	0.55	0.0007	0.02
Tachycardia^a^	354 (81.2)	168 (86.2)	624 (89.3)	0.13	0.23	0.0001	0.76	0.44	0.49
Tachypnea^a^	368 (84.4)	174 (89.2)	583 (83.4)	0.11	0.05	0.66	0.44	0.34	0.92
Hypotension^a^	368 (84.4)	111 (56.9)	265 (37.9)	<0.0001	<0.0001	<0.0001	0.004	0.42	<0.0001
Hypoxia^a^	101 (23.2)	79 (40.5)	227 (32.5)	<0.0001	0.04	0.0008	0.94	0.96	0.99
Laboratory findings, no. (%)									
Leukocytosis^a^	237 (54.4)	85 (43.6)	229 (32.8)	0.01	0.005	<0.0001	0.14	0.003	<0.0001
Leukopenia^a^	36 (8.3)	6 (3.1)	21 (3.0)	0.02	0.96	<0.0001	0.99	0.99	0.92
Increased creatinine^a^	107 (24.5)	4 (2.1)	1 (0.1)	<0.0001	0.009	<0.0001	0.97	0.99	0.68
Thrombocytopenia^a^	40 (9.2)	0 (0.0)	1 (0.1)	<0.0001	1.0	<0.0001	0.43	0.99	0.70
Hyperbilirubinemia^a^	44 (10.1)	1 (0.5)	1 (0.1)	<0.0001	0.39	<0.0001	0.82	0.94	0.68
Hyperlactatemia^a^	168 (38.5)	70 (35.9)	320 (45.8)	0.53	0.01	0.02	0.99	0.99	0.98
Coagulopathy^a^	67 (15.4)	2 (1.0)	1 (0.1)	<0.0001	0.12	<0.0001	0.87	0.86	0.67
Classification, no. (%)									
Sepsis	108 (24.8)	99 (50.8)	584 (83.6)	<0.0001	<0.0001	<0.0001	0.93	0.78	0.68
Septic shock	328 (75.2)	96 (49.2)	115 (16.5)						

^a^Hyperthermia: temperature >38 °C, hypothermia: temperature <36 °C, tachycardia: heart rate >90/min, tachypnea: respiratory rate >20/min, hypotension: systolic blood pressure <90, mean arterial pressure <65 or systolic blood pressure decrease >40 mmHg from baseline, hypoxia: oxygen requirement to maintain oxygen saturation >90%, leukocytosis: WBC count >12 × 10^9^/L, leucopenia: white blood cell count <4 × 10^9^/L, increased creatinine: creatinine increase >176.8 mmol/L, thrombocytopenia: platelet count <100 × 10^9^/L, hyperbilirubinemia: bilirubin >34.2 mmol/L, hyperlactatemia: lactate >2 mmol/L, coagulopathy: international normalized ratio (INR) >1

## Results

### Patients baseline characteristics

At the time of identification, patients in the intervention phases I and II were less likely to have hypothermia, altered mental status, hypotension, hypoxemia, leukopenia, elevated creatinine, thrombocytopenia, hyperbilirubinemia and coagulopathy compared to patients in the pre-intervention phase (Table 1). These differences in symptoms and signs, and key laboratory findings between the three phases suggest that the implementation of multifaceted interventions including sepsis e-alert and SRT was associated with earlier identification of sepsis.

### Propensity scores

The balancing effect of the generated propensity scores is shown in Table 1. When adjusted to propensity scores, all baseline characteristics were balanced.

### Interventions during the course of treatment

**Fig. 1:**
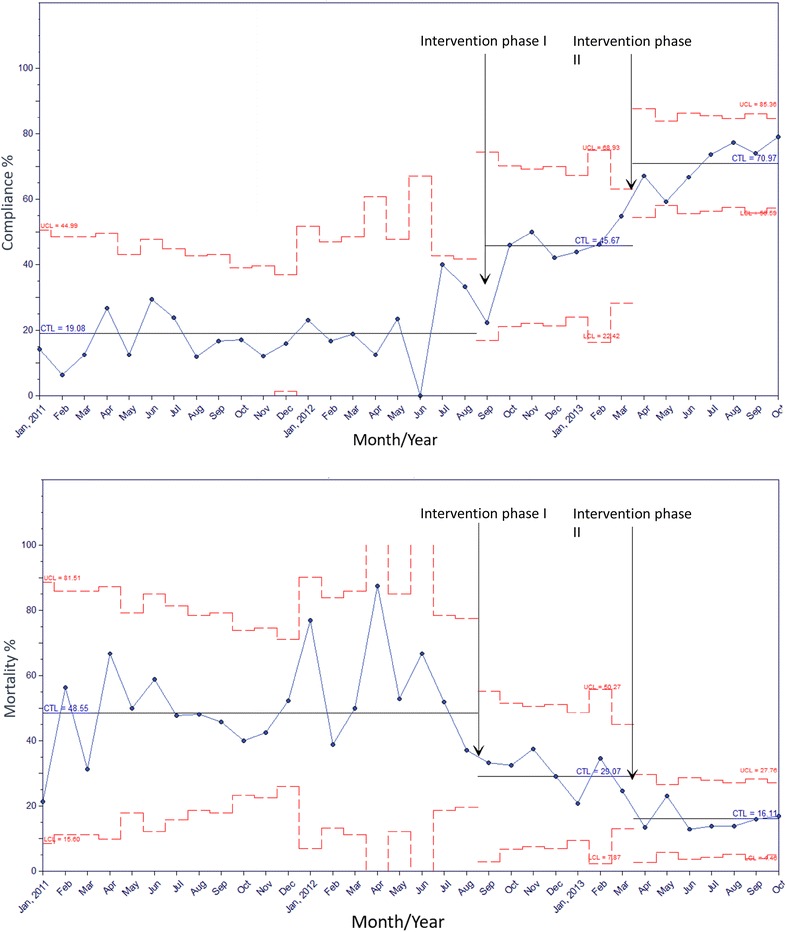
Control chart with *upper* and *lower* control limits of the overall compliance with the resuscitation bundle and the mortality in the three phases. The two arrows on the chart show the time of initiation of the intervention phases I and II

Intervention phases I and II were associated with improvement in most measured processes of sepsis care. The frequency of measuring lactate (at any time and within 6 h) and the time to obtaining lactate all improved. Similarly the frequency of obtaining blood cultures (at any time and before antibiotics) and the time to blood cultures improved. Antibiotics administration within 3 h and the time to antibiotics followed a similar pattern. In patients with persistent hypotension or lactate >4 mmol/L, CVP ≥8 mmHg and ScvO_2_ of ≥70% were achieved more often (Table 2). As a result, the overall compliance with 4-element bundle increased from 19.3 to 44.6 to 69.4% (*p* values <0.0001 after adjustment to propensity scores) (Table 2; Fig. 1).

**Table 2:** Sepsis-related interventions during the treatment period for patients with sepsis or septic shock in the three study phases: pre-intervention (A), intervention phase I (B) and intervention phase II (C)

Variable	Pre-intervention A	Intervention phase I B	Intervention phase II C	*p* value			*p* value after propensity scores adjustments		
All patients	*N* = 436	*N* = 195	*N* = 699	B vs. A	C vs. B	C vs. A	B vs. A	C vs. B	C vs. A
Lactate measured at any time, no. (%)	406 (93.1)	191 (97.9)	682 (97.6)	0.01	1.0	0.0003	0.02	0.72	0.009
Time to lactate (h), mean ± SD	9.3 ± 22.1	2.5 ± 5.3	1.1 ± 3.7	<0.0001	0.0008	<0.0001	0.002	0.0002	<0.0001
Lactate (mmol/L), mean ± SD	3.9 ± 3.6	3.3 ± 2.3	3.4 ± 5.5	0.01	0.93	0.03	0.76	0.28	0.26
Measure lactate within 6 h, no. (%)	270 (61.9)	168 (86.2)	663 (94.9)	<0.0001	<0.0001	<0.0001	<0.0001	<0.0001	<0.0001
Blood culture obtained at any time, no. (%)^a^	238 (54.6)	119/194 (61.3)	592/679 (87.2)	0.11	<0.0001	<0.0001	0.16	<0.0001	<0.0001
Time to blood culture (h), mean ± SD	2.4 ± 6.7	0.9 ± 1.4	0.9 ± 2.5	0.001	0.94	0.0008	0.02	0.84	0.0001
Blood culture before antibiotics, no. (%)	219 (50.2)	116 (59.5)	565 (80.8)	0.02	<0.0001	<0.0001	0.10	<0.0001	<0.0001
Antibiotics administered at any time, no. (%)^a^	266/269 (98.9)	136/137 (99.3)	439/448 (98.0)	1.0	0.47	0.55	0.97	0.45	0.87
Time to antibiotic administration (h), mean ± SD	4.8 ± 6.7	3.0 ± 3.6	1.9 ± 3.3	0.0004	0.002	<0.0001	0.002	0.002	0.01
Antibiotics within 3 h, no. (%)	295 (67.7)	149 (76.4)	625 (89.4)	<0.0001	<0.0001	<0.0001	0.01	0.0001	<0.0001

Patients with hypotension/lactate >4 mmol/L	*N* = 406	*N* = 149	*N* = 361						
Fluids delivered at any time, no. (%)^a^	399 (98.3)	148 (99.3)	344 (95.3)	0.69	0.02	0.02	0.51	0.15	0.51
MAP raised and remained >65 mmHg, no. (%)	88/399 (22.1)	53/148 (35.8)	229/344 (67.8)	0.001	<0.0001	<0.0001	0.02	0.50	0.09
Vasopressors administered, no. (%)	299/311 (96.1)	91/95 (95.8)	101/115 (87.8)	1.0	0.04	0.002	0.72	0.07	0.05
Fluids (≥20 mL/kg) and vasopressors in the first 6 h, no. (%)	364/412 (88.4)	144/153 (94.1)	324/370 (87.6)	0.04	0.03	0.74	0.04	0.08	0.85
Compliance with the 4 elements bundle	84 (19.3)	87 (44.6)	485 (69.4)	<0.0001	<0.0001	<0.0001	<0.0001	<0.0001	<0.0001

Patients with persistent hypotension/lactate >4 mmol/L	*N* = 333	*N* = 115	*N* = 209						
CVP ≥ 8 mmHg achieved, no. (%)									
Within 6 h	71/358 (19.8)	24/121 (19.8)	64/245 (26.1)	0.99	0.18	0.07	0.47	0.0002	<0.0001
Within 24 h	190 (57.2)	51 (44.4)	98 (46.9)	0.002	0.69	<0.0001	0.06	0.004	0.66
After 24 h	42 (12.7)	6 (5.2)	7 (3.4)						
Time to CVP (h), mean ± SD	16.3 ± 21.8	11.3 ± 10.4	8.1 ± 9.5	0.01	0.05	<0.0001	0.25	0.02	0.005
ScvO_2_ ≥ 70% achieved, no. (%)									
Within 6 h	65/358 (18.2)	19/121 (15.7)	56/245 (22.9)	0.53	0.11	0.16	0.69	<0.0001	0.0006
Within 24 h	106 (31.9)	26 (22.6)	78 (37.3)	0.03	0.57	0.006	0.003	<0.0001	0.31
After 24 h	37 (11.1)	5 (4.4)	7 (3.6)						
Time to ScvO_2_ (h) mean ± SD	20.8 ± 52.1	9.8 ± 13.7	8.2 ± 12.7	<0.0001	<0.0001	<0.0001	0.41	0.40	0.09

^a^Patients who had cultures or received antibiotics prior to meeting sepsis and septic shock criteria were not included

^a^20 mL/kg of crystalloid fluids *CVP* central venous pressure, *MAP* mean arterial pressure, *ScvO*_*2*_ central venous oxygen saturation. Denominators are equal to ‘*N*’ in all three phases unless otherwise specified

### Outcomes

Intervention phase II was associated with reduction in crude hospital mortality and ICU LOS (Fig. 1; Table 3). When adjusted to propensity score, intervention phase II was associated with a significant reduction in hospital mortality [adjusted odds ratio (aOR) 0.71, 95% CI 0.58–0.85, *p* = 0.003] and in the need for mechanical ventilation (aOR 0.45, 95% CI 0.37–0.55, *p* < 0.0001). In addition, intervention phase II was associated with reduction in ICU LOS and hospital LOS for all patients as well as ICU LOS for survivors (Table 3).

**Table 3:** Outcomes among patients with sepsis or septic shock in the three study phases: pre-intervention (A), intervention phase I (B) and intervention phase II (C) with adjustment to propensity scores

Variable	Pre-intervention A	Intervention phase I B	Intervention phase II C	Propensity scores adjusted OR or correlation coefficient* (95% CI, *p* value)		
All patients	*N* = 436	*N* = 195	*N* = 699	B vs. A	C vs. B	C vs. A
Hospital mortality, no. (%)	208 (47.7)	60 (30.8)	118 (16.9)	0.73 (0.48–1.09, 0.13)	0.78 (0.51–1.17, 0.23)	0.71 (0.58–0.85, 0.003)
ICU LOS (days), mean ± SD	13.3 ± 17.4	8.6 ± 8.3	5.1 ± 11.4	−4.49 (−7.39 to −1.59, 0.002)*	−1.54 (−3.38 to 0.29, 0.09)*	−2.72 (−3.92 to −1.52, <0.0001)*
Mechanical ventilation, no. (%)	265 (60.8)	54 (27.7)	78 (11.2)	0.33 (0.22–0.49, <0.0001)	0.64 (0.41–0.99, 0.05)	0.45 (0.37–0.55, <0.0001)
ICU LOS among survivors (days), mean ± SD	15.9 ± 19.5	11.9 ± 10.4	8.2 ± 13.8	−5.88 (−11.44 to −0.22, 0.04)*	−1.05 (−5.22 to 3.10, 0.61)*	−3.55 (−6.13 to −0.98, 0.007)*
Hospital LOS (days), mean ± SD	29.3 ± 33.9	25.7 ± 40.9	15.3 ± 23.5	−1.37 (−8.28 to 5.53, 0.70)*	−8.42 (−13.29 to −3.54, 0.0007)*	−5.35 (−7.76 to −2.94, <0.0001)*
Hospital LOS among survivors (days), mean ± SD	28.1 ± 30.8	24.7 ± 37.7	20.6 ± 28.6	−1.76 (−12.09 to 8.57, 0.73)*	−6.22 (−16.9 to 4.48, 0.25)*	−3.45 (−7.83 to 0.92, 0.12)*

*LOS* length of stay, * correlation coefficient (95% CI, *p* value)

## Discussion

Our study demonstrates that the implementation of a multifaceted intervention including a sepsis e-alert with an SRT was associated with improvement in the care process of sepsis management; improvement in the timeliness of lactate measurement, obtaining of blood cultures, antibiotic administration and achievement of CVP and ScvO_2_ targets and reduction in the need for mechanical ventilation and reduction in hospital mortality and LOS.

Quality improvement projects that implemented sepsis resuscitation bundle [43] have been shown to reduce mortality in several settings [20, 22, 26]. However, the effect varied considerably and was often modest, particularly when instruction-based interventions were used [22]. Our study showed a substantial improvement when a multifaceted approach was used that included an e-alert with SRT. The use of e-alert with SRT exemplifies the use of automation and forcing function in improving adherence to the best practice.

Timely recognition of sepsis is key to improving its management. A sepsis e-alert that is activated as soon as abnormal values are entered in the EHR may serve as a decision support system that bypasses the shortcomings of human processes. In addition, electronic screening for sepsis is more reliable and sustainable than paper-based screening tools as demonstrated in other settings [44]. Our data show that the e-alert identified patients with sepsis cases at earlier stage, which is a major aim of the project. However, early recognition may raise concerns about workload increase and overtreatment. However, in our project, physicians reviewed all cases before initiating treatment; thus reducing the risk of overtreatment. Additionally, it is our belief that the implications of overtreatment, if exists, are small compared to the substantial advantage of timely sepsis management.

Improving compliance with evidence-based time-sensitive therapies may be achieved by using condition-specific teams, as shown with acute myocardial infarction [45], stroke [46] and trauma teams [47]. Such teams are more focused, knowledgeable and experienced thus reducing care variation and increasing reliability. Only few studies have examined SRT implementation. A prospective cohort study examined the impact of ‘team’ vs. ‘non-team’ models on implementing sepsis bundle in multiple Asian countries [21]. In the non-team model, ED physicians completed the bundle in the ED as a standard care [21]. In the team model, the implementation was championed by intensivists with the bundle completed in the ICU and was associated with a greater improvement in the compliance [21]. Another study examined the impact of daily auditing with weekly feedback and SRT activation in patients admitted to the medical ICU with sepsis or septic shock [26]. The compliance rate with the sepsis resuscitation bundle increased from 12.7% at baseline to 37.7 and 53.7% during the weekly feedback and SRT activation periods, respectively (*p* < 0.001) [26]. The overall hospital mortality rates were 30.3, 28.3 and 22.0% during baseline, weekly feedback and SRT team periods, respectively (*p* = 0.03) [26]. In this study, the SRT was involved only in managing patients admitted to the medical ICU and required restructuring the existing ICU service, without adding new manpower [26].

There have been a heated debate about using CVP and ScvO_2_ as treatment goals in patients with sepsis; a debate that manifested intensely among our staff during the course of our project. Three recent trials of early goal-directed therapy demonstrated that routine use of central hemodynamic and oxygen-saturation monitoring compared to their use at-physician discretion did not result in better outcomes [40–42] resulted in removal of these elements from the sepsis resuscitation bundle [48]. Our data show significant improvement in outcomes even with modest increase in the compliance with the CVP and ScvO_2_ elements, supporting the notion that the key to improving sepsis outcome lies mainly in the early recognition, early fluid administration and early antibiotic therapy [48]. Why bundle compliance fell short of 100% even in the SRT phase?. An important reason is that the 2008 SCC bundle accounts for compliance achievement rather than attempting-to-achieve therapeutic goals within the recommended time [49], which has been revised in the 2012 version. Other reasons may include the complexity and high number of interventions, involvement of multiple players, resistance to change and the long waiting time to access care in our ED.

Our study has demonstrated that early identification and management of sepsis was associated with a reduction the need for mechanical ventilation and vasopressor therapy illustrating the notion that organ dysfunction in sepsis is preventable by early treatment. Some physicians prefer a slower pace of fluid resuscitation than what has been recommended in the SSC guidelines with the perception that a faster approach may precipitate pulmonary edema. While this concern may be valid in selected patients with very poor cardiovascular status, our study shows that early fluid resuscitation is generally associated with reduction in the need for mechanical ventilation.

SRT implementation, which is complex and intensive, should take into account factors that facilitate its sustainability, such as senior and clinical leadership engagement, evidence credibility, staff involvement and training, infrastructure and alignment with the organization strategic aim and culture [50]. SRT implementation, monitoring of compliance and comparing of outcomes require considerable infrastructure, resources and costs, which should be considered before adopting such an intervention. Nevertheless, we believe that the SRT may be a cost-effective intervention given the significant reduction in LOS, although a proper cost-effectiveness analysis is needed.

Our results should be interpreted in light of the study strengths and limitations. Strengths include the prospective design and the use of data to drive improvement. Limitations include being a single-center study and the pre–post nature. The study was limited to the ED intervention and did not include septic patients who were referred from the wards or were already in the ICUs. Because baseline compliance was low, the room for improvement was large. Therefore, it is possible that impact of this intervention could be less in centers with better performance at baseline. In addition, the e-alert system was built internally using the existing EHR and based on the available data and has not been validated externally. However, this will be likely the case for most similar projects, and our data can be used to support the concept of e-alert rather that a particular system. It remains to be studied whether designing an e-alert for sepsis screening using the new Sepsis-3 criteria would be superior to e-alerts based on the SIRS criteria like ours. Due to the nature of the database, we evaluated the timing of antibiotic therapy, but not antibiotic adequacy or adherence to local guidelines. Our data show learning effect, as reflected by improvement in compliance preceding the actual date of each phase on the control chart. Preparation to any intervention, whether the e-alert or the SRT was discussed among the staff who were invited to provide their input into the project; therefore, it is not surprising to see this learning effect. Such phenomenon is not unusual in quality improvement projects. We think that having this learning effect or ‘contamination’ is acceptable as improving the care was the final goal; however, this may have underestimated the difference between the phases. ​Nevertheless, the intervention phases I and II are distinct phases; with the second phase has manpower implications; therefore, we analyzed them as separate phases. Due to the nature of the SSC database, certain data elements were not available such as age, gender, comorbidities and severity of illness. Although our analysis suggests that the propensity scores were able to balance the baseline differences, the availability of such data elements may have further improved their performance. The possibility of ascertainment bias (detection of milder cases) is inherent in studies that implement screening systems. However, the differences remained significant after adjustment to propensity scores.

 In conclusion, the implementation of a multifaceted intervention including a sepsis e-alert system with SRT was associated with improvement in care processes of sepsis and septic shock and reduced need for mechanical ventilation as well as reduced mortality and LOS.

## Additional file

**Additional file 1: Figure S1.** Sepsis Response Team Clinical Pathway. **Figure S2**. Sepsis Response Team Checklist. **Table S1**. Sepsis Response Team Policy and Procedures.

## Abbreviations

CI: confidence interval
CPOE: computerized physician order entry
CVP: central venous pressure
e-alert: electronic alert
ED: emergency department
EHR: electronic health record
ICU: intensive care department
LOS: length of stay
PDSA: plan, do, study and act
SAS: statistical analysis software
ScvO_2_: central venous oxygen saturation
SRT: sepsis response team
SSC: surviving sepsis campaign
