# Supplementary material for: The impact of a multifaceted intervention including sepsis electronic alert system and sepsis response team on the outcomes of patients with sepsis and septic shock
Source: Ann Intensive Care. 2017 May 30;7:57. doi: 10.1186/s13613-017-0280-7 (PMC5449351; doi:10.1186/s13613-017-0280-7)
Supplement: Supplementary file 1 — Additional file 1: Figure S1. Sepsis Response Team Clinical Pathway. Figure S2. Sepsis Response Team Checklist. Table S1. Sepsis Response Team Policy and Procedures. [file 13613_2017_280_MOESM1_ESM.doc]

## TABLE OF CONTENTS

## Figure S1: Sepsis Response Team Clinical Pathway

## Figure S2: Sepsis Response Team Checklist

## Table S1: Sepsis Response Team Policy and Procedures

##

## e-Figure 2: Sepsis Team Checklist

##
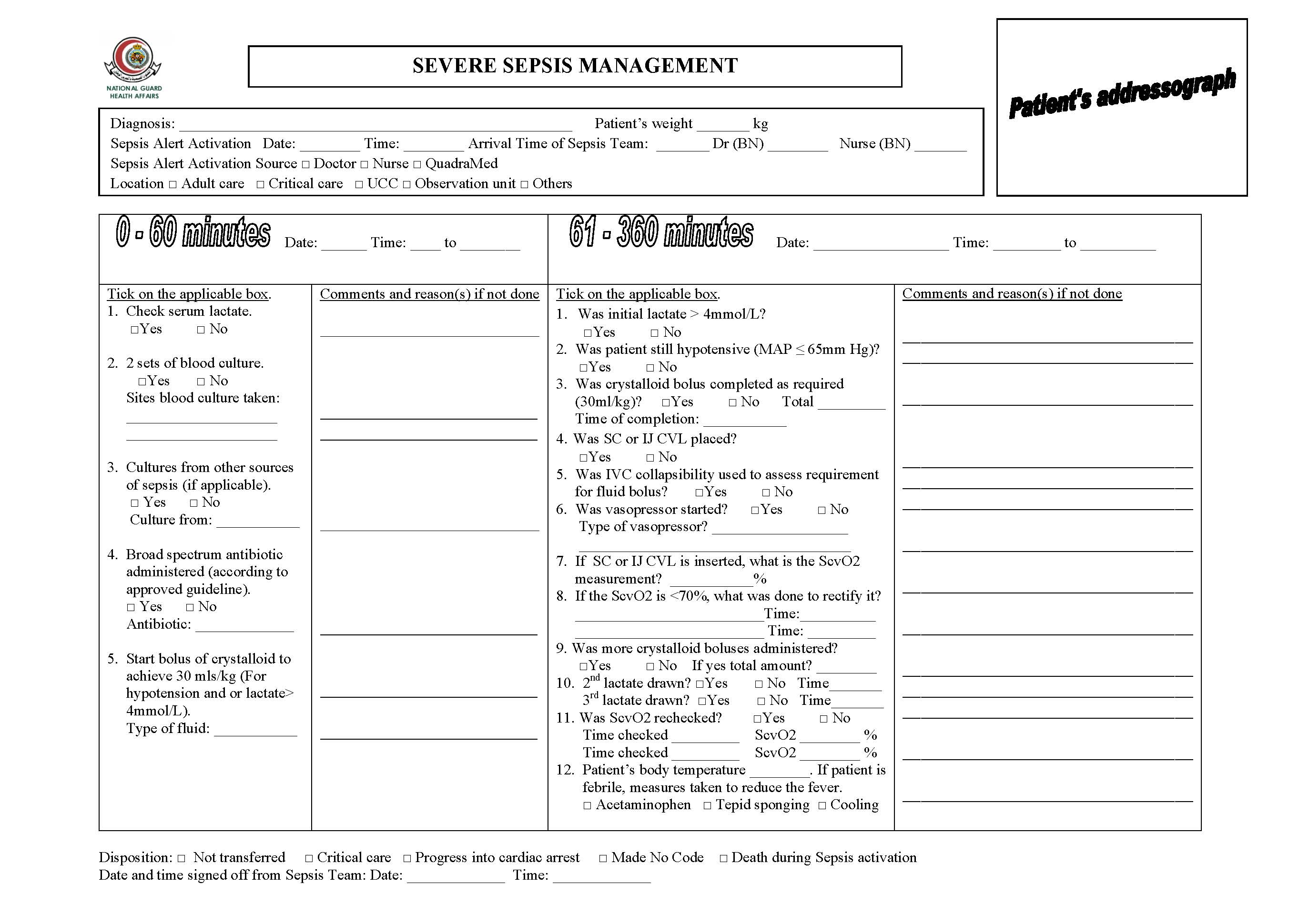


**e Table 1: SEPSIS RESPONSE TEAM**
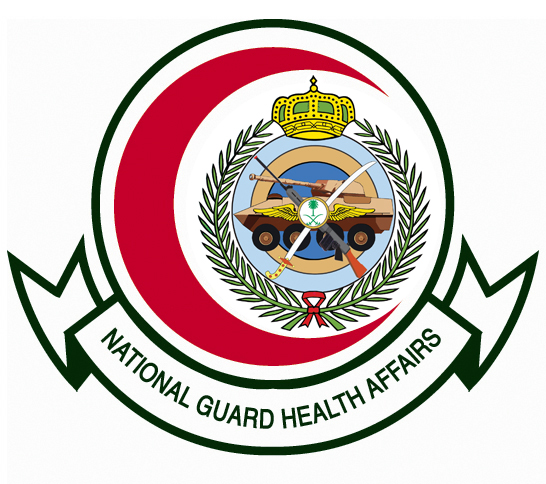
 **POLICY AND PROCEDURES**

**1. STATEMENT OF PURPOSE:** The aim of this document is to describe the policy and procedure for the activation, implementation and role of the Sepsis Response Team (SRT).

**2. APPLICABILITY:** This APP applies to all staff who may activate the SRT to assess a patient, or are a member of the SRT. The SRT responds to adult patients at King Abdulaziz Medical City- Central Region upon the activation of the SRT.

**3. SRT COMPOSITION**: the team core members are

#### One ICU fellow/ Staff Physician/ assistant consultant

- One Critical Care Nurse

Other healthcare providers who are part of the team include

- The ICU Consultant covering ED– Critical Care area for activations from the ED or ICU consultant covering CCRT for activations from other areas.
- The bedside Registered Nurse
- The area Charge Nurse
- The MRP or the MRP designee.
- The Pharmacist of the activating area.
- The Respiratory Therapist/ Technician of the activating area, if needed

**4. SRT ACTIVATION:**

- - - The SRT is activated by the bedside nurse when he/she receives a “SEVERE SEPSIS ALERT” in the nursing work list.
    - The SRT can be also activated by the bedside healthcare provider(s) including the registered nurse and/or physician when a patient demonstrates signs of severe sepsis or septic shock.
    - Activation of the SRT does not require a prior order or an explanatory progress note.
    - The SRT can be activated 24/7.
    - SRT will not be activated for patients who are labeled with “NO CODE”' status.
    - The SRT is activated by the activating nurse/physician calling the SRT pagers. These pagers are carried by the SRT physician and nurse at all time.
    - Upon activation of the SRT, the bed-side registered nurse will arrange for:
      - Notification of the attending physician or designee that SRT has been activated for their patient.
      - Communication with the SRT members regarding the patient's condition using the SBAR method.
      - Notification of the Respiratory Therapist/ Technician covering the activating area to be present at the bedside if needed.
      - Preparation of the patient's clinical record for review.
- The attending physician or designee may be requested to be present at the bedside as a key member for the SRT.

**6. SRT RESPONSIBILITIES**

- The SRT members will arrive within fifteen (15) minutes from the time of activation.
- SRT activation authorizes the SRT members to access the patient's clinical record, assess the patient's clinical status, write physician orders as required, perform procedures as needed, arrange patient transfers to a higher level of care, if necessary, access the crash cart, discuss patient’s status with family, if needed, recommend a change in Code Status.
- The SRT members will record and document the assessment, interventions and recommendations on the SBAR form**.**
- The primary healthcare team will continue to be responsible for the care of the patient, as long as his/her condition does not require transfer to a higher level of care. In the latter case, the SRT will make the arrangements required for this transfer in collaboration with the attending physician or ED consultant.
- The SRT will communicate with primary attending physician team/ ED consultant regarding the further care if the patient is critically ill but does not meet severe sepsis criteria**.**
- The SRT will provide non-judgmental feedback, education and support to the primary healthcare team.
- The SRT will follow-up the patient for up to 6 hours. This is the usual period needed to implement the Sepsis Resuscitation bundle and to initiate and make a plan for Sepsis Management bundle.
- If the patient becomes under ICU service before the end of this period, the SRT will ensure the implementation of the sepsis resuscitation and management bundles.
- If the patient needs critical care beyond the 6 hour period, he/ she will be referred to the ICU team.

**Specific responsibilities of the SRT Physician**

- Responds with the SRT nurse to activations and follow-ups.
- Receives handoff from the activating nurse or physician.
- Assesses the patient for severe sepsis or septic shock.
- Initiates/ advocates the implementation of Sepsis– Early Goal-Directed Therapy and sepsis management bundle (such as appropriate antibiotics are ordered/given within 30 minutes of arrival if not already given).
- Communicates with the covering ICU consultant for patients with severe sepsis who have the following conditions:
  - Septic shock (mean arterial blood pressure <65 mmHg after adequate fluid resuscitation and requirement for vaso-active medications).
  - Acute respiratory failure: PaO_2_/FiO_2_ <150, Respiratory rate >30/ min or <8/min, PCO2 >60 with pH <7.30, requirement of invasive or noninvasive ventilation.
  - Need for central line insertion.
  - The patient is candidate for NO CODE status.
- Determines the final disposition of the patient after discussion with the admitting service.
- W**rites a note in the medical record and progress reports for follow-ups.**
- Assures completion of the SRT Data Collection Tools.
- Provides educational feedback to other team members.

**Specific responsibilities of the SRT Nurse**

- Responds to all SRT Activation.
- Receives a report from the previous shift.
- Implements algorithms and protocols.
- Assists with acute resuscitation, line placement and monitoring.
- Provides educational in-services to bedside Registered Nurses
- Documents data for every patient.
- Maintains SRT data collection
